# Full aspiration technique using a 7-Fr double-pigtail stent for endoscopic ultrasound-guided pancreatic fluid drainage of a pancreatic pseudocyst

**DOI:** 10.1055/a-2779-3430

**Published:** 2026-02-17

**Authors:** Shunsuke Omoto, Mamoru Takenaka, Akihiro Yoshida, Kae Fukunishi, Hidekazu Tanaka, Yoriaki Komeda, Masatoshi Kudo

**Affiliations:** 1 326473 Departments of Gastroenterology and Hepatology, Kindai University Faculty of Medicine, Sakai, Japan

Endoscopic ultrasound–guided pancreatic fluid drainage (EUS-PFD) is an established treatment for pancreatic pseudocysts 1 . To prevent pseudocyst infection caused by stent occlusion, multiple plastic stents are often placed 2 . However, when fistula dilation is difficult, placing two stents is technically challenging, and a single 7-Fr stent has been associated with an increased risk of stent occlusion 3 . Large pseudocysts have also been associated with stent migration, especially when fluid drainage is insufficient 4 5 . Achieving reliable drainage with fewer plastic stents remains challenging. We developed a “full aspiration technique” to place a single 7-Fr double-pigtail stent after aspirating all cystic fluid before stent release ( Video 1 ).

EUS-guided drainage of a postoperative pancreatic pseudocyst using the “full aspiration technique,” involving complete cystic fluid aspiration before stent deployment to prevent occlusion and migration. EUS, endoscopic ultrasound.Video 1

**Fig. 1:**
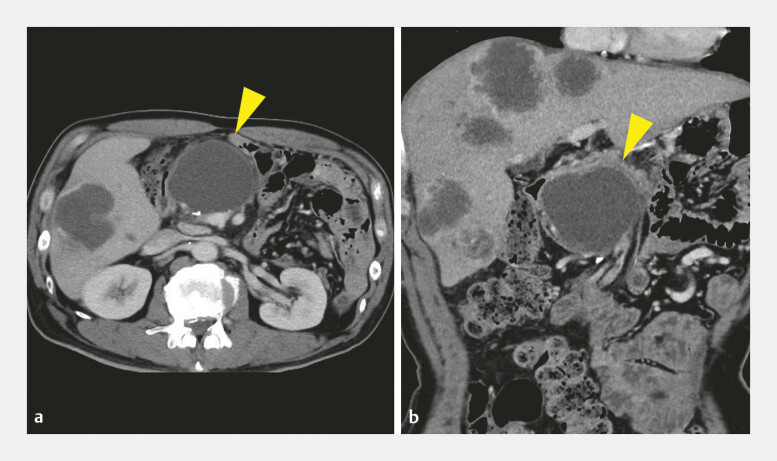
**a** and **b** Pre-procedural CT showing a 66-mm postoperative pancreatic pseudocyst (yellow arrowhead) in the pancreatic head. CT, computed tomography.

A 54-year-old man, who had undergone distal pancreatectomy for pancreatic cancer, presented with epigastric pain. Laboratory tests showed elevated white blood cell count and C-reactive protein. MDCT revealed a 66-mm cystic lesion in the pancreatic head, diagnosed as a pancreatic pseudocyst ( Fig. 1 ).

**Fig. 2:**
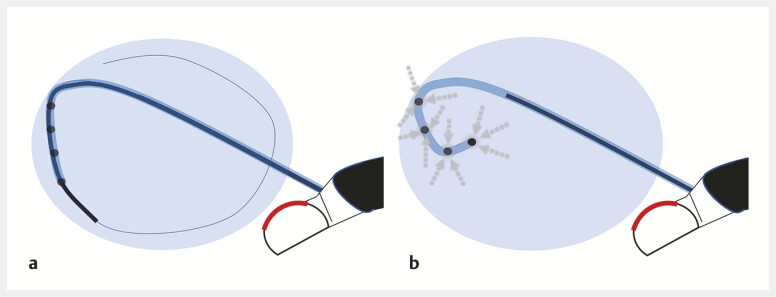
**a** Placement of a double-pigtail catheter into the cyst cavity. **b** After the partial withdrawal of the inner sheath, the guidewire is removed, and the cystic fluid is fully aspirated through the inner guiding catheter lumen (full aspiration technique).

**Fig. 3:**
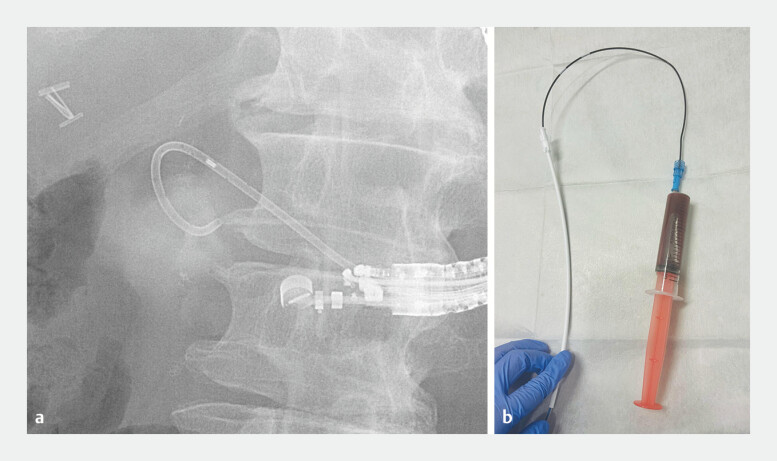
A fluoroscopic image **a** showing the partial deployment of a 7-Fr double-pigtail stent, and a procedural field image **b** demonstrating the aspiration of 85 mL of cystic fluid through the inner guiding catheter lumen.

**Fig. 4:**
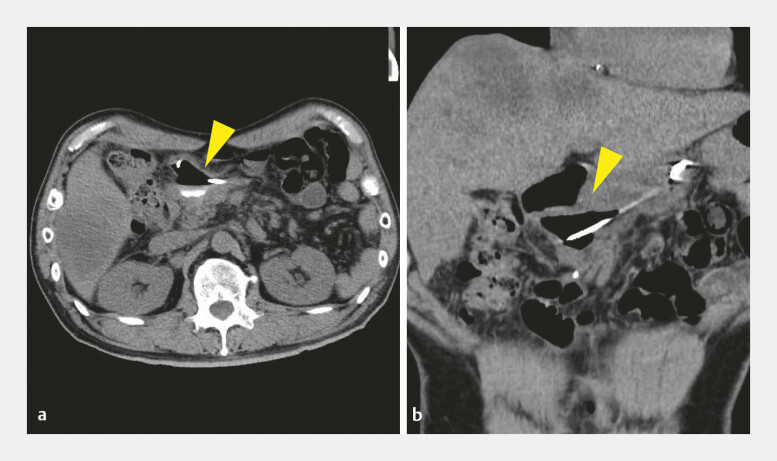
**a** and **b** Post-procedural CT demonstrating the complete resolution of the pancreatic pseudocyst (yellow arrowhead). CT, computed tomography.

EUS-PFD was performed. After puncture with a 19G EZ Shot 3 needle (Olympus, Tokyo, Japan), bloody fluid was aspirated. As the cyst partially shrank and shifted, tract dilation became difficult. A drill dilator (Tornus ES, Olympus, Tokyo, Japan) was used, but tract dilation remained challenging. The cavity was re-expanded by injecting 20 mL of saline through a catheter and then dilated with a 4-mm REN balloon (Kaneka Medix, Osaka, Japan). A 7-Fr/7-cm double-pigtail stent (Through & Pass, Gadelius Medical, Tokyo, Japan) was deployed halfway, the guidewire was removed, and 85 mL of fluid was aspirated ( Fig. 2 and Fig. 3 ). The stent was then released into the stomach. The procedure was completed without adverse events, and post-procedural computed tomography confirmed complete resolution ( Fig. 4 ). This novel technique allows complete aspiration of the cystic fluid before stent release, reducing the risk of stent occlusion and migration by shrinking the cyst.

Endoscopy_UCTN_Code_TTT_1AS_2AJ
